# *catena*-Poly[[bis­(thio­cyanato-κ*N*)iron(II)]-bis­(μ-dipyrazin-2-yl di­sulfide-κ^2^ *N* ^4^:*N* ^4′^)]

**DOI:** 10.1107/S1600536813021958

**Published:** 2013-08-10

**Authors:** Susanne Wöhlert, Inke Jess, Christian Näther

**Affiliations:** a Institut für Anorganische Chemie, Christian-Albrechts-Universität Kiel, Max-Eyth-Strasse 2, 24118 Kiel, Germany

## Abstract

In the title compound, [Fe(NCS)_2_(C_8_H_6_N_4_S_2_)_2_]_*n*_, the Fe^II^ cation is coordinated by two terminal *N*-bonded thio­cyanate anions and four bridging *N*:*N*′-bridging dipyrazin-2-yl di­sulfide ligands in an octa­hedral geometry. The Fe^II^ cations are connected *via* bridging 4,4′-di­pyrazine ligands into chains along the *b*-axis direction. The asymmetric unit consists of one Fe^II^ cation located on position with site symmetry 2/*m*, one thio­cyanate anion located on a mirror plane and one di­sulfide ligand located on a twofold rotation axis.

## Related literature

For general background to this work, see: Wriedt & Näther (2011 ▶). For a description of the Cambridge Structural Database, see: Allen (2002 ▶).
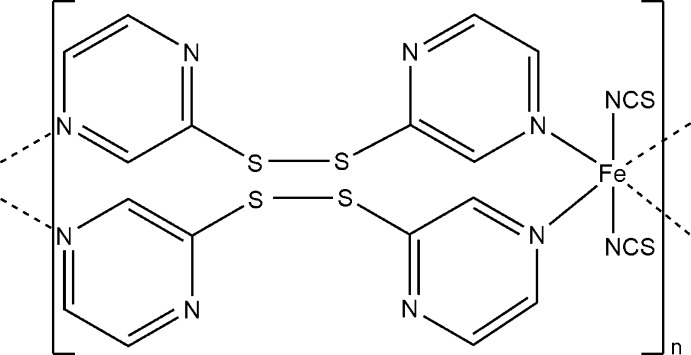


## Experimental

### 

#### Crystal data

[Fe(NCS)_2_(C_8_H_6_N_4_S_2_)_2_]
*M* *_r_* = 616.59
Orthorhombic, 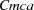

*a* = 19.053 (1) Å
*b* = 8.0559 (5) Å
*c* = 16.1952 (9) Å
*V* = 2485.8 (2) Å^3^
*Z* = 4
Mo *K*α radiation
μ = 1.14 mm^−1^
*T* = 293 K
0.11 × 0.08 × 0.05 mm

#### Data collection

Stoe IPDS-2 diffractometer
Absorption correction: numerical (*X-SHAPE* and *X-RED32*; Stoe & Cie, 2008 ▶) *T*_min_ = 0.782, *T*_max_ = 0.902
7765 measured reflections
1242 independent reflections
1077 reflections with *I* > 2σ(*I*)
*R*_int_ = 0.035

#### Refinement

*R*[*F* ^2^ > 2σ(*F* ^2^)] = 0.051
*wR*(*F* ^2^) = 0.113
*S* = 1.15
1242 reflections
86 parameters
H-atom parameters constrained
Δρ_max_ = 0.69 e Å^−3^
Δρ_min_ = −0.34 e Å^−3^

### 

Data collection: *X-AREA* (Stoe & Cie, 2008 ▶); cell refinement: *X-AREA*; data reduction: *X-AREA*; program(s) used to solve structure: *SHELXS97* (Sheldrick, 2008 ▶); program(s) used to refine structure: *SHELXL97* (Sheldrick, 2008 ▶); molecular graphics: *XP* in *SHELXTL* (Sheldrick, 2008 ▶) and *DIAMOND* (Brandenburg, 2012 ▶); software used to prepare material for publication: *XCIF* in *SHELXTL*.

## Supplementary Material

Crystal structure: contains datablock(s) I, global. DOI: 10.1107/S1600536813021958/nr2047sup1.cif

Structure factors: contains datablock(s) I. DOI: 10.1107/S1600536813021958/nr2047Isup2.hkl

Additional supplementary materials:  crystallographic information; 3D view; checkCIF report

## Figures and Tables

**Table:** 

Fe1—N1	2.061 (4)
Fe1—N10	2.273 (3)

**Table:** 

N1^i^—Fe1—N1	180.00 (18)
N1^i^—Fe1—N10	89.81 (11)
N1—Fe1—N10	90.19 (11)
N10—Fe1—N10^ii^	90.62 (13)
N10—Fe1—N10^iii^	89.38 (13)

Symmetry codes: (i) 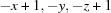; (ii) 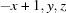; (iii) 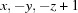.

**Fig. 1.**
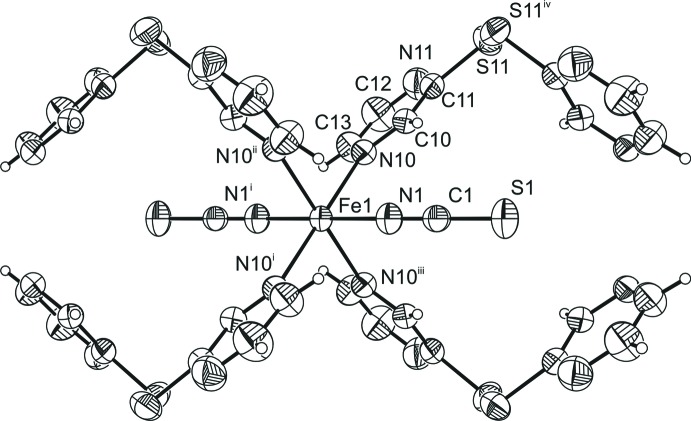
Crystal structure of the title compound with labeling and displacement ellipsoids drawn at the 50% probability level. Symmetry code: i = -x + 1, -y, -z + 1; ii = x, -y, -z + 1; iii = -x + 1, y, z; iv = x, -y + 1, -z + 1.

**Fig. 2.**
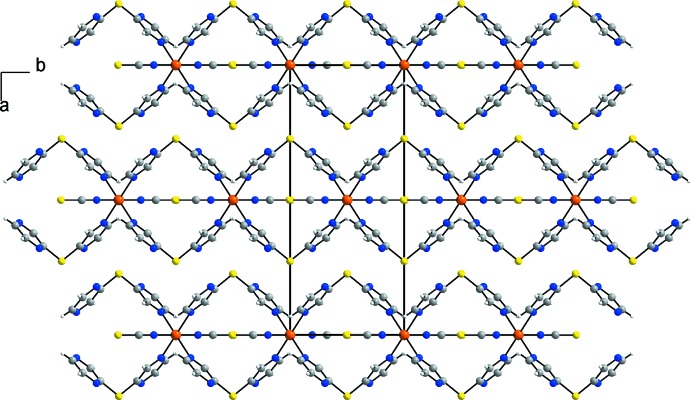
View of the chains that elongate in the direction of the crystallographic b axis.

## supplementary crystallographic information

### 1. Comment

This work is part of a project on the synthesis and characterization of new coordination compounds based on transition metal thiocyanates and different N-donor ligand (Wriedt & Näther, 2011). Crystals of the title compound were obtained by accident in the reaction of iron(II) sulfate heptahydrate with potassium thiocyanate and 2-chloropyrazine. To identify the product of this reaction a structure determination was performed.

In the crystal structure of the title compound each iron(II) cation is octahedrally coordinated by two terminal *N*-bonded thiocyanato anions and four bridging dipyrazine-disulfide ligands that has accidently formed in the reaction (Fig. 1 and Tab. 1). The Fe—NCS distances of 2.061 (4) Å and the Fe—N(dipyrazine-disulfide) distances of 2.273 (3) Å are in the normal range (Tab. 1). The Fe^II^ cations are located on position 2/m, the thiocyanato anions on a mirror plane and the dipyrazine-disulfide ligands on a 2-fold axis (Fig. 1). The iron(II) cations are linked into chains by the dipyrazine-disulfide ligands that elongate in the direction of the crystallographic *b*-axis (Fig. 2). It must be noted that according to a search in the CCDC database such compounds with dipyrazine-disulfide are unknown (ConQuest Ver. 1.14 2012, Allen, 2002).

### 2. Experimental

FeSO_4_.7H_2_O and 2-chloropyrazine were obtained from Sigma Aldrich. KNCS was obtained from Alfa Aesar. 0.6 mmol (168.8 mg) FeSO_4_.7H_2_O, 1.2 mmol (118.5 mg) KNCS and 0.15 mmol (13.2 µL) 2-chloropyrazine were reacted with 1 mL H_2_O in a closed test-tube at 120°C for three days. On cooling red block-shaped single crystals of the title compound has formed.

### 3. Refinement

All H atoms were located in difference map but were positioned with idealized geometry and were refined isotropic with *U*_iso_(H) = 1.2 *U*_eq_(C) of the parent atom using a riding model with C—H = 0.93 Å.

### Crystal data

**Table:**

[Fe(NCS)_2_(C_8_H_6_N_4_S_2_)_2_]	*F*(000) = 1248
*M**_r_* = 616.59	*D*_x_ = 1.648 Mg m^−^^3^
Orthorhombic, *C**m**c**a*	Mo *K*α radiation, λ = 0.71073 Å
Hall symbol: -C 2bc 2	Cell parameters from 7765 reflections
*a* = 19.053 (1) Å	θ = 3.0–26.0°
*b* = 8.0559 (5) Å	µ = 1.14 mm^−^^1^
*c* = 16.1952 (9) Å	*T* = 293 K
*V* = 2485.8 (2) Å^3^	Block, red
*Z* = 4	0.11 × 0.08 × 0.05 mm

### Data collection

**Table:**

Stoe IPDS-2 diffractometer	1242 independent reflections
Radiation source: fine-focus sealed tube	1077 reflections with *I* > 2σ(*I*)
Graphite monochromator	*R*_int_ = 0.035
ω scan	θ_max_ = 26.0°, θ_min_ = 3.0°
Absorption correction: numerical (*X-SHAPE* and *X-RED32*; Stoe & Cie, 2008)	*h* = −23→21
*T*_min_ = 0.782, *T*_max_ = 0.902	*k* = −9→9
7765 measured reflections	*l* = −19→17

### Refinement

**Table:**

Refinement on *F*^2^	Primary atom site location: structure-invariant direct methods
Least-squares matrix: full	Secondary atom site location: difference Fourier map
*R*[*F*^2^ > 2σ(*F*^2^)] = 0.051	Hydrogen site location: inferred from neighbouring sites
*wR*(*F*^2^) = 0.113	H-atom parameters constrained
*S* = 1.15	*w* = 1/[σ^2^(*F*_o_^2^) + (0.0413*P*)^2^ + 5.3642*P*] where *P* = (*F*_o_^2^ + 2*F*_c_^2^)/3
1242 reflections	(Δ/σ)_max_ < 0.001
86 parameters	Δρ_max_ = 0.69 e Å^−^^3^
0 restraints	Δρ_min_ = −0.34 e Å^−^^3^

### Special details

**Table:**

Geometry. All esds (except the esd in the dihedral angle between two l.s. planes) are estimated using the full covariance matrix. The cell esds are taken into account individually in the estimation of esds in distances, angles and torsion angles; correlations between esds in cell parameters are only used when they are defined by crystal symmetry. An approximate (isotropic) treatment of cell esds is used for estimating esds involving l.s. planes.
Refinement. Refinement of F^2^ against ALL reflections. The weighted R-factor wR and goodness of fit S are based on F^2^, conventional R-factors R are based on F, with F set to zero for negative F^2^. The threshold expression of F^2^ > 2sigma(F^2^) is used only for calculating R-factors(gt) etc. and is not relevant to the choice of reflections for refinement. R-factors based on F^2^ are statistically about twice as large as those based on F, and R- factors based on ALL data will be even larger.

### Fractional atomic coordinates and isotropic or equivalent isotropic displacement parameters (Å^2^)

**Table:**

	*x*	*y*	*z*	*U*_iso_*/*U*_eq_	
Fe1	0.5000	0.0000	0.5000	0.0384 (3)	
N1	0.5000	0.1936 (5)	0.5832 (3)	0.0507 (10)	
C1	0.5000	0.3240 (5)	0.6134 (3)	0.0397 (10)	
S1	0.5000	0.50646 (16)	0.65366 (9)	0.0640 (4)	
N10	0.41517 (13)	0.1291 (3)	0.42501 (17)	0.0428 (6)	
C10	0.37695 (15)	0.2524 (4)	0.4557 (2)	0.0445 (7)	
H10	0.3819	0.2818	0.5109	0.053*	
C11	0.32962 (17)	0.3380 (4)	0.4062 (2)	0.0490 (8)	
C12	0.3600 (2)	0.1830 (6)	0.2972 (2)	0.0713 (11)	
H12	0.3561	0.1563	0.2415	0.086*	
C13	0.4057 (2)	0.0943 (5)	0.3454 (2)	0.0579 (9)	
H13	0.4309	0.0073	0.3219	0.070*	
N11	0.32128 (18)	0.3053 (4)	0.3271 (2)	0.0649 (9)	
S11	0.27348 (5)	0.50102 (12)	0.43778 (8)	0.0687 (3)	

### Atomic displacement parameters (Å^2^)

**Table:**

	*U*^11^	*U*^22^	*U*^33^	*U*^12^	*U*^13^	*U*^23^
Fe1	0.0371 (5)	0.0304 (4)	0.0478 (5)	0.000	0.000	−0.0021 (4)
N1	0.053 (2)	0.039 (2)	0.060 (2)	0.000	0.000	−0.0075 (19)
C1	0.037 (2)	0.041 (2)	0.041 (2)	0.000	0.000	0.0057 (19)
S1	0.0814 (10)	0.0398 (7)	0.0709 (9)	0.000	0.000	−0.0098 (6)
N10	0.0386 (14)	0.0393 (13)	0.0505 (15)	0.0010 (11)	0.0018 (12)	0.0033 (11)
C10	0.0367 (15)	0.0400 (16)	0.0567 (18)	0.0007 (13)	−0.0023 (15)	0.0000 (14)
C11	0.0394 (17)	0.0375 (16)	0.070 (2)	−0.0023 (13)	−0.0072 (16)	0.0005 (15)
C12	0.080 (3)	0.079 (3)	0.055 (2)	0.011 (2)	−0.010 (2)	−0.005 (2)
C13	0.060 (2)	0.057 (2)	0.056 (2)	0.0099 (18)	−0.0037 (18)	−0.0035 (17)
N11	0.065 (2)	0.0616 (19)	0.068 (2)	0.0059 (16)	−0.0192 (17)	0.0015 (16)
S11	0.0492 (5)	0.0527 (5)	0.1041 (8)	0.0138 (4)	−0.0205 (5)	−0.0146 (6)

### Geometric parameters (Å, º)

**Table:**

Fe1—N1^i^	2.061 (4)	C10—C11	1.390 (4)
Fe1—N1	2.061 (4)	C10—H10	0.9300
Fe1—N10	2.273 (3)	C11—N11	1.318 (5)
Fe1—N10^ii^	2.273 (3)	C11—S11	1.769 (3)
Fe1—N10^iii^	2.273 (3)	C12—N11	1.323 (5)
Fe1—N10^i^	2.273 (3)	C12—C13	1.371 (5)
N1—C1	1.158 (6)	C12—H12	0.9300
C1—S1	1.608 (5)	C13—H13	0.9300
N10—C10	1.328 (4)	S11—S11^iv^	2.015 (2)
N10—C13	1.332 (5)		
			
N1^i^—Fe1—N1	180.00 (18)	C10—N10—C13	116.5 (3)
N1^i^—Fe1—N10	89.81 (11)	C10—N10—Fe1	122.1 (2)
N1—Fe1—N10	90.19 (11)	C13—N10—Fe1	121.1 (2)
N1^i^—Fe1—N10^ii^	89.81 (11)	N10—C10—C11	120.7 (3)
N1—Fe1—N10^ii^	90.19 (11)	N10—C10—H10	119.6
N10—Fe1—N10^ii^	90.62 (13)	C11—C10—H10	119.6
N1^i^—Fe1—N10^iii^	90.19 (11)	N11—C11—C10	122.7 (3)
N1—Fe1—N10^iii^	89.81 (11)	N11—C11—S11	110.9 (3)
N10—Fe1—N10^iii^	89.38 (13)	C10—C11—S11	126.4 (3)
N10^ii^—Fe1—N10^iii^	180.0	N11—C12—C13	122.3 (4)
N1^i^—Fe1—N10^i^	90.19 (11)	N11—C12—H12	118.9
N1—Fe1—N10^i^	89.81 (11)	C13—C12—H12	118.9
N10—Fe1—N10^i^	180.00 (11)	N10—C13—C12	121.8 (4)
N10^ii^—Fe1—N10^i^	89.38 (13)	N10—C13—H13	119.1
N10^iii^—Fe1—N10^i^	90.62 (13)	C12—C13—H13	119.1
C1—N1—Fe1	164.1 (4)	C11—N11—C12	116.0 (3)
N1—C1—S1	179.0 (4)	C11—S11—S11^iv^	106.45 (13)

Symmetry codes: (i) −*x*+1, −*y*, −*z*+1; (ii) −*x*+1, *y*, *z*; (iii) *x*, −*y*, −*z*+1; (iv) *x*, −*y*+1, −*z*+1.
